# Periodontitis and Pancreatic Cancer Risk: A Systematic Review, Meta-Analysis, and Trial Sequential Analysis

**DOI:** 10.3390/jcm15083154

**Published:** 2026-04-21

**Authors:** Kareelend Andreina Segura Cueva, Andrea Bermúdez Velásquez, Carlos Andrés Guim Martínez, Luis Chauca-Bajaña, Leonardo Javier Siguencia Suárez, Byron Velásquez Ron, Carlos E. Cuevas-Suárez, Abigailt Flores-Ledesma, Alejandro Ismael Lorenzo-Pouso, Andrea Ordoñez Balladares

**Affiliations:** 1 College of Dentistry, University of Guayaquil, Guayaquil 090101, Ecuador; kareelend.segurac@ug.edu.ec (K.A.S.C.);; 2 School of Dentistry, Universidad Católica de Santiago de Guayaquil (UCSG), Guayaquil 090101, Ecuador; 3 Carrera de Odontología, Department Prosthesis Research, Universidad de Las Américas (UDLA), Quito 170102, Ecuador; byron.velasquez@udla.edu.ec; 4 Dental Materials Laboratory, Academic Area of Dentistry, Autonomous University of Hidalgo State, San Agustín Tlaxiaca 42160, Mexico; cecuevas@uaeh.edu.mx; 5 Dental Materials and Biomaterials Laboratory, Faculty of Stomatology, Meritorious Autonomous University of Puebla, Puebla 72570, Mexico; 6 Oral Medicine, Oral Surgery and Implantology Unit (MedOralRes), Faculty of Medicine and Dentistry, University of Santiago de Compostela, 15782 Santiago de Compostela, Spain; 7 College Dentistry, Universidad Bolivariana del Ecuador, Durán 092402, Ecuador

**Keywords:** periodontitis, pancreatic neoplasms, risk factors, tooth loss, inflammation

## Abstract

**Introduction**: Pancreatic cancer is one of the most lethal malignancies worldwide, and its incidence continues to rise. Periodontitis, a highly prevalent chronic inflammatory disease, has been linked to several systemic conditions, including a potential increase in pancreatic cancer risk. However, the available epidemiological evidence remains heterogeneous and fragmented. **Objective**: To evaluate whether periodontitis is associated with an increased risk of pancreatic cancer through a systematic review and meta-analysis of observational studies. **Materials and Methods**: A comprehensive search was conducted in PubMed, EMBASE, Web of Science, Scopus, the Cochrane Library, ClinicalTrials.gov, and the WHO regional databases, following PRISMA guidelines. Cohort, case–control, and cross-sectional studies assessing periodontitis through clinical parameters, radiographic measures, or tooth loss—and reporting pancreatic cancer risk (HR, RR, or OR)—were included. Risk of bias was assessed using the Newcastle–Ottawa Scale. Random-effects meta-analyses, meta-regressions, leave-one-out sensitivity analyses, influence diagnostics, publication bias assessment, and Trial Sequential Analysis (TSA) were performed. **Results**: Eight observational studies (primarily cohort designs) (*n* = 476,245 participants) met the inclusion criteria. Periodontitis was associated with an increased risk of pancreatic cancer (pooled HR = 1.56; 95% CI: 1.28–1.89), with moderate heterogeneity (I^2^ = 55.5%). Sensitivity and influence analyses confirmed the robustness of the estimate. TSA showed a consistent trend, although the cumulative evidence remains insufficient for a definitive conclusion. **Conclusions**: Observational evidence suggests a modest statistical association between periodontitis and pancreatic cancer risk. However, the absolute risk increase is very small, and Trial Sequential Analysis indicates that cumulative evidence remains insufficient to establish causality or to support preventive or clinical recommendations. Further large-scale prospective studies with standardized periodontal assessments are required.

## 1. Introduction

Pancreatic cancer remains one of the most lethal malignancies worldwide due to its late diagnosis, clinically silent course, and the limited effectiveness of available therapeutic options [1]. The global burden of the disease has increased over recent decades, driven by demographic, environmental, and metabolic changes [2]. This scenario has prompted the investigation of potentially modifiable risk factors to guide earlier prevention and detection strategies [3].

In parallel, periodontitis, a highly prevalent chronic inflammatory disease, has emerged as a relevant systemic determinant due to its ability to generate persistent inflammation and oral dysbiosis [4]. This condition affects the tooth-supporting tissues and is characterized by a complex interaction between periodontal pathogens, the host immune response, and microbiome alterations [5]. The systemic impact of periodontitis has been linked to multiple non-communicable chronic diseases, prompting a reconsideration of its role beyond the oral cavity [6].

Multiple epidemiological studies have demonstrated associations between periodontitis, tooth loss, and the development of various types of cancer [7]. A plausible explanation is that chronic inflammation plays a fundamental role in carcinogenesis, as suggested by biological models and classic reviews [8]. The continuous activation of inflammatory pathways, the production of immune mediators, and alterations in the tissue microenvironment may contribute to tumor initiation and progression [9].

Recent research has identified dysbiosis of the oral microbiome as a relevant factor in both digestive diseases and gastrointestinal cancers [10]. The oral cavity acts as a reservoir of microorganisms capable of translocating, altering the immune response, or modifying metabolic processes with systemic impact [11]. Pathogens such as *Porphyromonas gingivalis* and *Aggregatibacter actinomycetemcomitans* can induce sustained pro-inflammatory responses and modulate pathways related to cellular transformation [12]. Oral dysbiosis has also been shown to influence the microbial composition of other segments of the digestive tract [13].

In the specific case of pancreatic cancer, several cohort studies have suggested that periodontal disease increases its risk in adults [14]. Similarly, national registry-based studies have shown consistent associations between periodontitis, tooth loss, and a higher incidence of pancreatic cancer [15]. Additional studies across different populations have also observed a positive relationship between poor oral health and this type of cancer [16].

Beyond epidemiological observations, microbiome research has identified oral microbial profiles associated with an increased risk of pancreatic cancer [17]. Consistently, serological studies have reported that elevated antibody levels against certain oral bacteria are associated with an increased risk of the disease [18]. Experimental evidence and specialized reviews have proposed biological mechanisms that explain the potential link between oral pathogens, systemic inflammation, and pancreatic carcinogenesis [19,20].

This systematic review and meta-analysis aims to examine the potential relationship between periodontitis and pancreatic cancer risk by compiling and synthesizing data from observational studies, as well as assessing the strength and consistency of the association and the factors that may explain its heterogeneity.

## 2. Materials and Methods

### 2.1. Protocol and Registration

**Figure 1:**
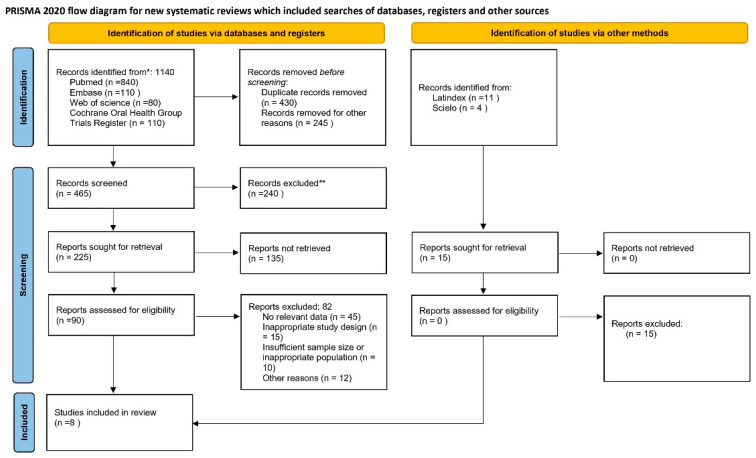
Flowchart of selected studies. * Records identified through database searching and other sources. ** Records excluded after title and abstract screening because they did not meet the predefined inclusion criteria.

A predefined protocol was developed for the search and data extraction procedures in accordance with the PRISMA guidelines [21]. The protocol was prospectively registered in the PROSPERO database (CRD420251244556). The PRISMA flow diagram is presented in Figure 1, and the completed PRISMA checklist is provided as Supplementary Materials (Table S1). The final electronic database search was conducted in October 2025.

### 2.2. Focused Question

Do adults with periodontitis have a higher risk of developing pancreatic cancer compared with those without periodontitis?

P (Population): Adults from the general population or patients receiving healthcare services, evaluated for their periodontal status.

I (Intervention/Exposure): Presence of periodontitis (diagnosed clinically, radiographically, or through a history of tooth loss attributable to periodontal disease).

C (Comparison): Individuals without periodontitis or with healthy/mild periodontal status.

O (Outcome): Incidence or risk of developing pancreatic cancer.

### 2.3. Information Sources and Search Strategy

The literature search was conducted using the Rayyan QCRI platform (Qatar Computing Research Institute, Doha, Qatar). Following PRISMA guidelines, a comprehensive and structured search strategy was applied across major international databases.

Medical Subject Headings (MeSH) and Emtree terms related to periodontal disease and pancreatic cancer were combined using Boolean operators. The core MeSH terms included: “Periodontitis”, “Periodontal Diseases”, “Alveolar Bone Loss”, “Tooth Loss”, “Pancreatic Neoplasms”, and “Pancreatic Cancer”. To ensure maximum sensitivity, additional keywords and free-text terms were incorporated, such as chronic periodontitis, gum disease, gingival inflammation, oral microbiota, oral pathogens, systemic inflammation, pancreatic carcinoma, and digestive system cancers.

The search covered the following electronic databases: MEDLINE (via PubMed), EMBASE (via OVID), Web of Science, Scopus, Cochrane Library, ClinicalTrials.gov, and the five regional WHO databases (AIM, LILACS, IMEMR, IMSEAR, WPRIM). The search was extended to the Conference Proceedings Citation Index to identify gray literature and non-indexed studies. No restrictions were applied regarding language or publication year.

To ensure completeness, we manually reviewed the reference lists of all included articles and of relevant narrative or systematic reviews. Authors’ personal collections and previously known relevant publications were also screened. This process was complemented by a targeted manual search of peer-reviewed journals specializing in periodontology, oncology, and epidemiology.

### 2.4. Eligibility Criteria

Inclusion Criteria:

1. Observational studies (cohort, nested case–control, or cross-sectional with risk estimates). Nested case–control studies derived from well-defined source populations were considered eligible, as they provide valid approximations of relative risk in rare outcomes such as pancreatic cancer.
2. Adults ≥ 18 years with documented periodontal assessment.
3. Periodontitis is diagnosed by clinical parameters (PD, CAL, BOP), radiographic bone loss, CDC/AAP criteria, or tooth loss due to periodontal disease.
4. Comparator group without periodontitis or with healthy/mild periodontal status.
5. Outcome reporting pancreatic cancer risk as RR, HR, OR, or with sufficient data for calculation.
6. Full-text peer-reviewed publications.
7. Articles published in English.

Exclusion Criteria:

1. Clinical trials, systematic reviews, meta-analyses, editorials, commentaries, or non-observational studies without risk estimates.
2. Animal studies, in vitro studies, or pediatric populations.
3. Studies evaluating general oral health without distinguishing periodontitis or using only “number of teeth” without periodontal attribution.
4. Studies assessing overall cancer without specific pancreatic cancer data, or unrelated benign/preneoplastic conditions.
5. Studies lacking sufficient information to obtain or reconstruct RR/HR/OR or without a clear comparator.
6. Duplicate publications (only the most complete version retained).

### 2.5. Study Selection Process and Data Extraction

Two independent reviewers (LC and AOB) screened and extracted data using a predesigned and standardized data extraction form. Any discrepancies between the two reviewers were resolved through consultation with a third reviewer (KS), who was blinded to the study hypothesis to minimize bias. Inter-reviewer agreement was evaluated at this stage using the kappa (κ) coefficient.

The following variables were extracted from each eligible study: first author, year of publication, country/region, study design, sample size, number of cases and controls, type of periodontal exposure (e.g., clinical periodontitis, tooth loss, periodontal attachment loss, oral microbiome markers), method of exposure assessment, outcome definition (pancreatic cancer incidence or mortality), effect size measures (HR, RR, OR) with 95% confidence intervals, adjustments for confounders, and any additional methodological characteristics relevant for risk-of-bias and heterogeneity analyses.

During the initial screening stage, titles and abstracts of all retrieved records were evaluated to identify potentially eligible studies. Full texts were then reviewed using predefined inclusion and exclusion criteria. When essential data were missing, unclear, or not directly extractable from the manuscript, attempts were made to contact the corresponding authors for clarification or additional information (Table 1).

### 2.6. Quality Assessment and Risk of Bias Evaluation

The risk of bias of the included observational studies was independently assessed by two reviewers (LC and AOB) using the Newcastle–Ottawa Scale (NOS) [25], following the recommendations for cohort study meta-analyses. The tool evaluates three methodological domains—Selection, Comparability, and Outcome Assessment assigning maximum scores of 4, 2, and 3 points, respectively. Discrepancies between reviewers were resolved by a third evaluator (BVR), who was blinded to the study objectives. Based on the scores obtained, each domain was operationally classified as low, moderate, or high risk. A graphical risk-of-bias matrix was subsequently generated to illustrate the overall distribution of methodological quality among the studies included. No study was excluded based on risk of bias, as all met an acceptable methodological standard for inclusion in the meta-analysis.

### 2.7. Certainty of Evidence Assessment (GRADE)

The certainty of evidence was assessed using the GRADE framework, considering risk of bias, inconsistency, indirectness, imprecision, and publication bias (Supplementary Table S2).

### 2.8. Statistical Analysis

All analyses were performed in R (version 4.3.1; R Foundation for Statistical Computing, Vienna, Europe, Austria) using the metafor, meta, dmetar, and ggplot2 packages. Individual effect estimates were transformed into log-hazard ratios (logHR) and their corresponding variances, and the pooled effect was calculated using random-effects models with the REML estimator, given the expected heterogeneity across studies. Heterogeneity was assessed using the Q statistic, τ^2^, and I^2^. Potential sources of heterogeneity were explored through univariable meta-regressions considering publication year, sample size, and geographic region. The influence of individual studies was examined through leave-one-out analyses and influence diagnostics. Publication bias was assessed using funnel plots, Egger’s test, and the trim-and-fill method. A cumulative meta-analysis was performed to evaluate the temporal stability of the effect, and subgroup analyses were conducted according to geographic region (Asia, Europe, United States). Finally, a modified Trial Sequential Analysis (TSA) was conducted to assess the sufficiency of the accumulated evidence and to rule out spurious findings due to random errors. A *p*-value < 0.05 was considered statistically significant.

## 3. Results

### 3.1. Qualitative Analysis

The search across databases and other sources identified 1155 records. After removing 430 duplicates and 245 records for other reasons, 465 studies remained for title and abstract screening, of which 240 were excluded. A total of 225 full-text articles were sought for retrieval, but 135 could not be obtained. Thus, 90 articles were assessed in full, and 82 were excluded due to lack of relevant data, inappropriate study design, insufficient sample size, or other reasons. Ultimately, 8 studies met all inclusion criteria and were incorporated into the systematic review and meta-analysis. The interviewer agreement was high k = 0.92 (92.20%).

### 3.2. Quality Assessment

**Figure 2:**
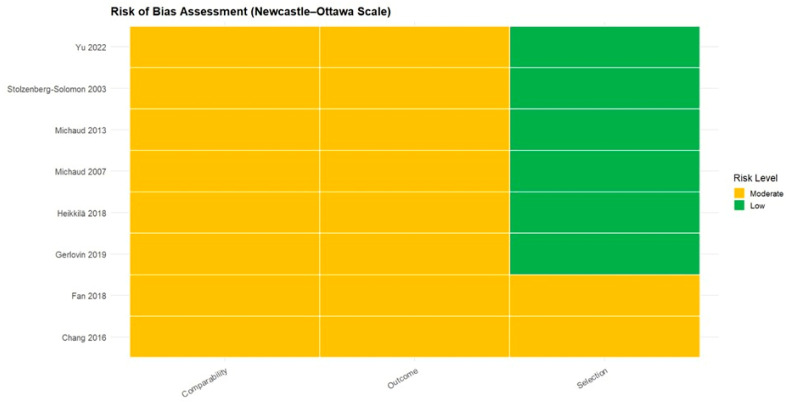
Risk of Bias Assessment Using the Newcastle–Ottawa Scale [14,15,16,17,18,22,23,24].

Quality assessment using the Newcastle–Ottawa Scale showed that all included studies demonstrated low risk of bias in the Selection domain, while the Comparability and Outcome domains were generally rated as moderate (Figure 2). No study was classified as high risk in any domain. Overall, the methodological quality of the included cohorts was acceptable, indicating that the evidence base for this review is reasonably robust.

### 3.3. Quantitative Sensitivity

#### Association Between Periodontitis and Pancreatic Cancer

**Figure 3:**
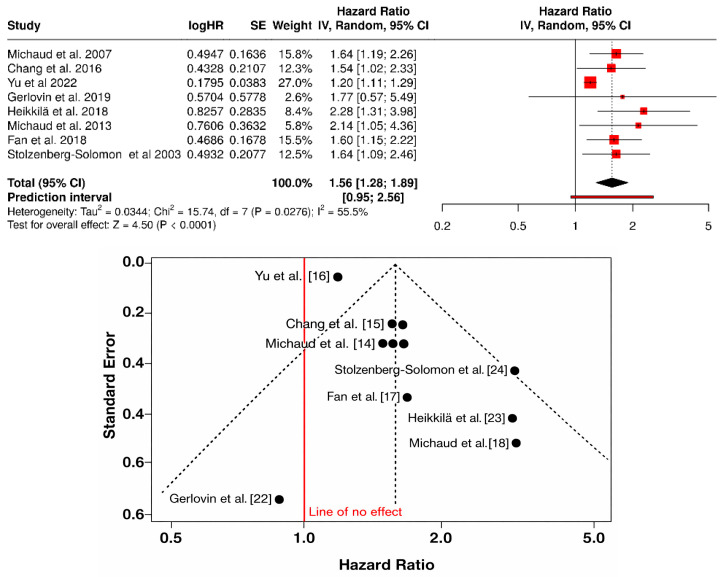
Forest plot and funnel plot of the association between periodontitis and pancreatic cancer risk [14,15,16,17,18,22,23,24].

Eight observational studies (primarily cohort designs) (total *n* = 476,245 participants) were included in the meta-analysis. The random-effects model showed that periodontitis was significantly associated with an increased risk of pancreatic cancer, with a pooled HR of 1.56 (95% CI: 1.28–1.89). Heterogeneity was moderate (I^2^ = 55.5%), while the prediction interval (0.95–2.56) indicated that future studies may range from no effect to stronger associations. These findings suggest a consistent relationship between periodontitis and elevated pancreatic cancer risk, although some variability across studies persists. Visual inspection of the funnel plot revealed slight asymmetry, primarily driven by studies with larger variances and smaller sample sizes. Most studies clustered around the pooled estimate, although a few peripheral points suggested potential publication bias or methodological heterogeneity (Figure 3).

### 3.4. Subgroup Analyses

**Figure 4:**
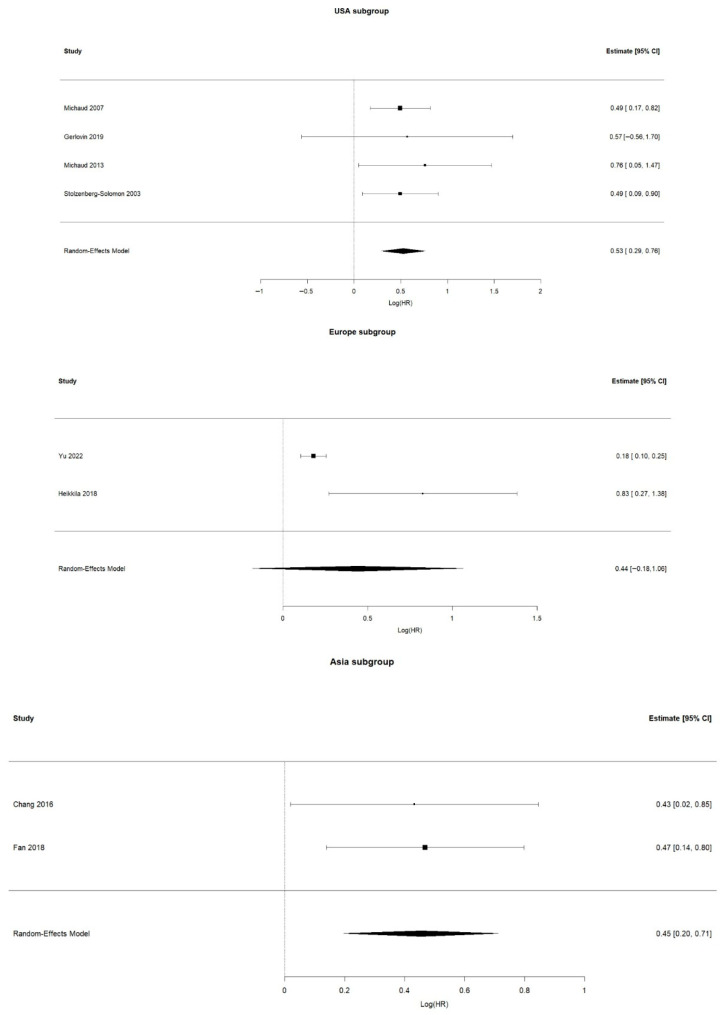
Subgroup forest plots by geographic region (Asia, USA, and Europe) [14,15,16,17,18,22,23,24].

The subgroup analyses stratified by geographic region revealed variations in the association between periodontitis and pancreatic cancer. In the Asian subgroup, both included studies showed a consistent and moderate positive association (pooled logHR = 0.45; 95% CI: 0.20–0.71). The U.S. subgroup demonstrated similar results, with a pooled logHR of 0.53 (95% CI: 0.29–0.76), indicating a stable and significant relationship across four independent cohorts. In contrast, the European subgroup showed greater variability, largely driven by differences in sample size and effect estimates between the studies, resulting in a non-significant pooled effect (logHR = 0.44; 95% CI: −0.18–1.06). Overall, the regional analyses confirm that the association between periodontitis and pancreatic cancer is strongest and most consistent in Asian and U.S. populations (Figure 4).

### 3.5. Trial Sequential Analysis

**Figure 5:**
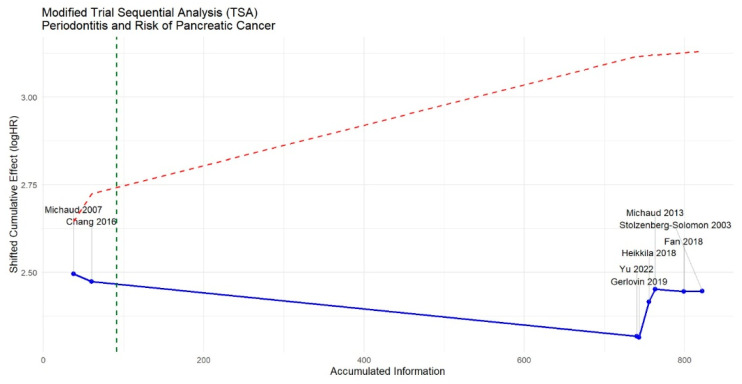
Trial Sequential Analysis evaluating the cumulative evidence for the association between periodontitis and pancreatic cancer [14,15,16,17,18,22,23,24].

Figure 5 presents the TSA, which evaluated the stability of the accumulated evidence. The cumulative effect curve showed initial fluctuations following the earliest studies (Michaud 2007 and Chang 2016) and progressively stabilized as larger and more recent cohorts were incorporated [14,15]. The direction of the cumulative evidence remained consistent with the main meta-analysis after inclusion of the remaining studies (Gerlovin 2019, Yu 2022, Heikkilä 2018, Fan 2018, Stolzenberg-Solomon 2003, and Michaud 2013) [16,17,18,22,23,24]. Although the overall trend supported a positive association, the cumulative Z-curve did not cross the TSA-adjusted monitoring boundaries, indicating that the accumulated evidence does not yet provide conclusive confirmation of a definitive effect. Therefore, the findings should be interpreted cautiously, as the observed association may still be influenced by random error. Additional well-powered prospective studies with standardized periodontal assessment are required to further clarify this relationship.

### 3.6. Meta-Regression of the Effect of Periodontitis on Pancreatic Cancer Risk

**Figure 6:**
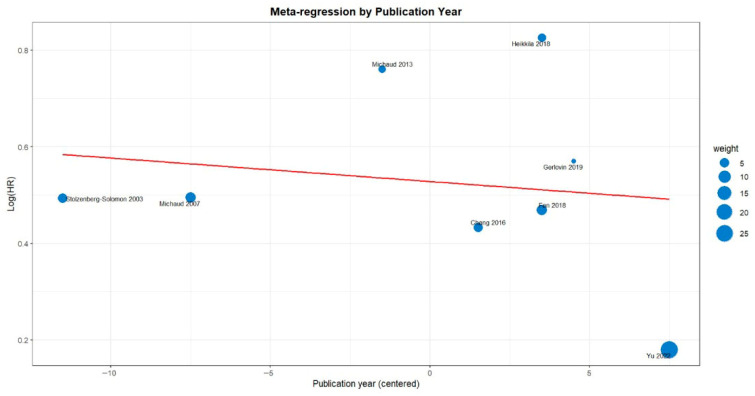

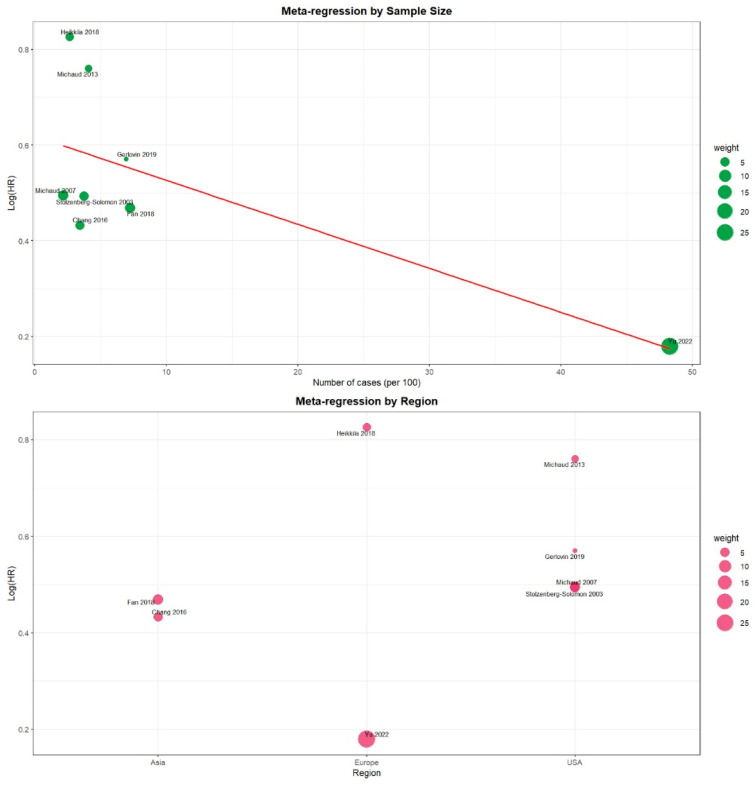
Meta-regressions by publication year, sample size, and geographic region [14,15,16,17,18,22,23,24].

Complementary meta-regressions were conducted to explore potential sources of heterogeneity (Figure 6). The meta-regression by publication year showed a slight negative slope, with older studies such as Stolzenberg-Solomon 2003 and Michaud 2007 reporting logHR values around 0.47–0.50, whereas the most recent large cohort (Yu 2022) showed the lowest estimate (logHR 0.18); however, the trend was not statistically significant [14,16,24]. The meta-regression by sample size displayed a clearer negative pattern, with smaller studies showing higher logHR values (up to 0.84 in Heikkilä 2018) and the largest cohort again showing the lowest effect (Yu 2022 0.18), though without significant effect modification [16,23]. Finally, the regional meta-regression showed no meaningful differences across Asia (0.43–0.46), Europe (0.18–0.84), and the United States (0.47–0.75), confirming that geographic region did not explain the observed heterogeneity.

### 3.7. Sensitivity Analysis

**Figure 7:**
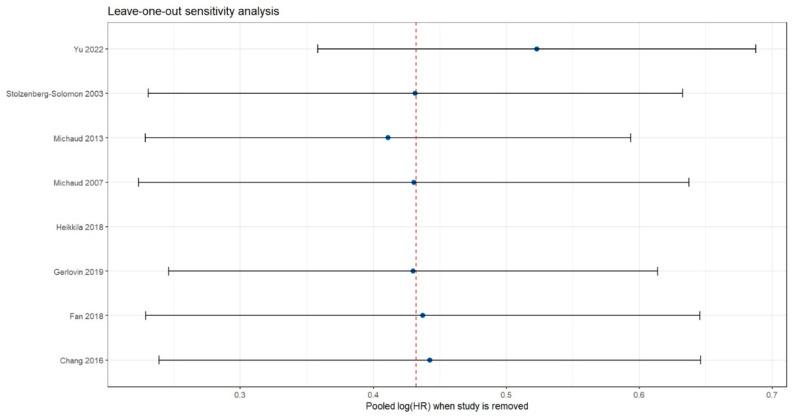
Leave-one-out sensitivity analysis evaluating the robustness of the pooled effect estimate [14,15,16,17,18,22,23,24].

The leave-one-out sensitivity analysis showed that removing each study sequentially resulted in pooled logHR values ranging approximately from 0.39 to 0.46, all of which remained close to the main model estimate (dashed red line 0.43). No individual study including the largest cohort (Yu 2022) or those with higher effect sizes such as Heikkilä 2018—produced a meaningful shift in the pooled effect or widened the confidence interval beyond the limits of the primary analysis [16,23]. These results indicate that no influential study was driving the association and confirm the robustness and stability of the meta-analytic findings (Figure 7).

### 3.8. Assessment of Publication Bias

**Figure 8:**
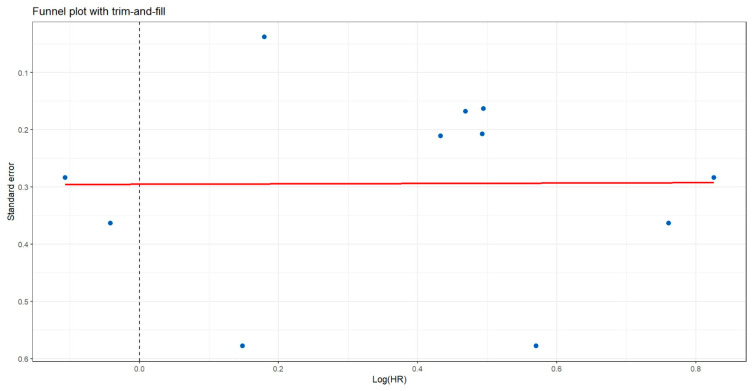
Publication Bias Assessment Using Trim-and-Fill Method.

The trim-and-fill analysis did not impute any missing studies, indicating no evidence of substantial publication bias. The funnel plot showed a largely symmetrical distribution of effect sizes, with logHR values ranging approximately from 0.05 to 0.85 across studies. The adjusted pooled effect (red line) remained essentially identical to the original estimate, suggesting that small-study effects or selective reporting are unlikely to have influenced the observed association between periodontitis and pancreatic cancer (Figure 8).

### 3.9. Influence Diagnostics and Study Impact Assessment

**Figure 9:**
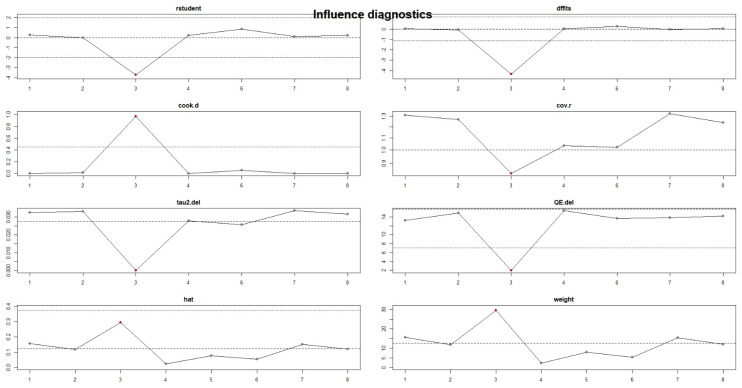
Influence Diagnostics for Individual Study Impact.

Influence diagnostics indicated that none of the studies exerted a disproportionate impact on the pooled effect (Figure 9). Studentized residuals remained within acceptable limits (all between approximately −3.5 and +1.5), and Cook’s distance values were uniformly low, with the largest peak (1.0) still below conventional influence thresholds. Similarly, leave-one-out changes in τ^2^ and Q statistics showed only minor fluctuations, and hat values remained stable (all < 0.35). Although one study showed slightly higher leverage across several indices, its influence remained insufficient to alter the pooled estimate. Overall, these results confirm that no single study unduly drove the association between periodontitis and pancreatic cancer.

## 4. Discussion

This systematic review and meta-analysis identified a modest statistical association between periodontitis and pancreatic cancer risk across Eight observational studies (primarily cohort designs). Nevertheless, this finding should be interpreted with caution, as the evidence base is limited in size, relies on heterogeneous definitions of periodontal exposure, and remains insufficient to establish a causal relationship. In historical cohorts, Michaud et al. (2007) showed that men with periodontitis had an elevated risk of pancreatic cancer [14], whereas Chang et al. (2016) confirmed this pattern in a large Asian population using ICD-9-based diagnoses [15]. Yu et al. (2022) contributed additional European evidence demonstrating that poor oral health was associated with modest yet significant risk increases, particularly among adults aged 50–70 years [16]. Stolzenberg-Solomon et al. (2003), in the ATBC study, also identified early in the century that severe tooth loss was linked to a higher risk of pancreatic cancer [24]. Collectively, these studies provide solid epidemiological evidence positioning periodontal health as a potential population-level determinant of pancreatic cancer. Other included studies offer complementary perspectives. Heikkilä et al. (2018) showed that clinical periodontitis is associated with greater pancreatic cancer mortality, suggesting a potential role in tumor progression [23]. Although Gerlovin et al. (2019) did not reach statistical significance, the observed pattern remains consistent with the direction of risk seen in other cohorts [22]. Metabacteriological evidence from Fan et al. (2018) indicates that periodontal taxa such as *Aggregatibacter actinomycetemcomitans* are significantly associated with increased risk [17]. This aligns with Michaud et al. (2013), who reported that elevated antibody levels against *P. gingivalis* increased pancreatic cancer risk in European cohorts [18]. Beyond classical epidemiology, recent studies provide a multilayered view of the periodontitis–pancreatic cancer connection. Farrell et al. (2012), Mitsuhashi et al. (2015), and RA Gaiser et al. (2019) documented altered oral microbial compositions in pancreatic cancer patients, particularly with overabundance of *P. gingivalis*, *Fusobacterium nucleatum*, and *A. actinomycetemcomitans* [26,27,28]. Meng et al. (2025) expanded this perspective by showing that oral microbial alterations precede pancreatic cancer diagnosis in large U.S. cohorts [29]. In parallel, strong mechanistic evidence demonstrates that periodontal pathogens possess procarcinogenic capabilities. Rubinstein et al. (2013) described how *F. nucleatum* promotes tumorigenesis via the FadA adhesin, which activates the E-cadherin–β-catenin pathway in epithelial cancer cells [30]. These findings were expanded by Abed et al. (2016), who showed that the Fap2 adhesin facilitates Fusobacterium tropism toward neoplastic tissue by binding specifically to Gal-GalNAc, enriching the tumor microenvironment [31]. Complementarily, Tan et al. (2022) demonstrated that *Porphyromonas gingivalis*, a key periodontal pathogen, enhances pancreatic cancer progression by activating neutrophil elastase derived from tumor-associated neutrophils, providing direct experimental evidence of the oncogenic potential of oral bacteria within the pancreas [32].

From an immunological and inflammatory perspective, strong evidence shows that periodontitis induces chronic systemic inflammation mediated by cytokines such as IL-6, TNF-α, and IL-1β. Foundational studies by Hajishengallis (2015) and Bartold & Van Dyke (2013) described how periodontal dysbiosis promotes persistent inflammatory responses with effects extending beyond the oral cavity [6,12], while Nibali et al. (2007) demonstrated significant increases in systemic inflammatory markers among individuals with severe periodontitis [33]. Likewise, comprehensive reviews by Meyer, Joshipura, Giovannucci, and Michaud (2008), along with Michaud’s 2017 update, showed that systemic inflammation and oral dysbiosis are linked to increased risk of multiple extraoral cancers, positioning periodontal health as a relevant indicator of chronic inflammation [7,34]. Similarly, Lamont and Hajishengallis (2015) reviewed how periodontal pathogens reprogram key immune pathways, promoting apoptosis evasion, sustained cellular proliferation, and immune tolerance mechanistically plausible processes in carcinogenesis [35]. The concept of the gum–gut–pancreas axis, discussed by Olsen and Yamazaki (2019), Lan Su et al. (2024), and Sun J (2020), proposes that oral bacteria may translocate or indirectly influence the gut microbiota, modulating mucosal immunity and fostering a pro-inflammatory microenvironment that could affect distant organs, including the pancreas [11,36,37]. This hypothesis is reinforced by Gaiser et al. (2019), who identified enrichment of oral-origin species in pancreatic cystic precursor lesions [28]. Finally, systematic reviews and meta-analyses such as those by Maisonneuve & Lowenfels (2015), Wang et al. (2022), and Márquez-Arrico et al. (2024) have reported consistent associations between periodontitis, tooth loss, and increased risk of pancreatic or other gastrointestinal cancers [38,39,40]. Together, these studies integrate epidemiological evidence, systemic inflammatory mechanisms, and recent microbial findings, reinforcing the overall coherence between periodontal disease and increased risk of digestive tract neoplasms. Methodologically, this meta-analysis demonstrated robustness: leave-one-out analyses showed stable effect estimates, influence diagnostics revealed no dominant studies, the trim-and-fill method detected no publication bias, and TSA suggested that although current evidence supports a positive association, additional large-scale studies with more standardized periodontal definitions are needed. Nonetheless, the consistency of the association across populations, diagnostic methods, bacterial taxa, antibodies, and experimental models strengthens the causal plausibility.

These findings carry potential clinical implications; however, they should be interpreted cautiously. Although periodontitis was associated with an increased relative risk of pancreatic cancer, the absolute risk increase remains small given the low incidence of the disease. Assuming a baseline annual incidence of approximately 12 cases per 100,000 individuals in the general population, the observed pooled HR of 1.56 would correspond to an estimated increase of about 6–7 additional cases per 100,000 individuals per year. This contextualization underscores that, despite statistical significance, the absolute effect at the population level is modest. Periodontal status could potentially be incorporated as a complementary component within multifactorial pancreatic cancer risk models, particularly among individuals with established risk factors such as smoking, obesity, diabetes, or familial predisposition. Nevertheless, it should not be considered a standalone screening tool, and current evidence does not justify changes in prevention or screening strategies. Future longitudinal studies with standardized periodontal definitions and integrated microbiome and inflammatory profiling are needed to clarify whether periodontitis acts as a causal factor, risk modifier, or marker of susceptibility.

From a clinical standpoint, these findings should not prompt immediate changes in screening practices but may encourage greater interdisciplinary awareness. Dentists and periodontists should recognize that chronic periodontal inflammation may reflect broader systemic inflammatory processes. Similarly, clinicians managing patients with multiple pancreatic cancer risk factors may consider periodontal health as part of a comprehensive risk profile. However, given the small absolute risk increase and the observational nature of the evidence, periodontal status should be interpreted as a potential marker rather than a direct causal factor.

Future research should prioritize the adoption of standardized and reproducible periodontal diagnostic criteria to improve comparability across epidemiological studies. The included cohorts demonstrated substantial variability in exposure assessment, ranging from clinical periodontal examinations and radiographic bone loss measurements to self-reported diagnoses and tooth loss proxies. Such heterogeneity complicates direct comparison between studies and may contribute to residual heterogeneity in pooled analyses. The implementation of internationally recognized diagnostic frameworks—such as the CDC/AAP surveillance case definitions or the 2018 Classification of Periodontal and Peri-Implant Diseases and Conditions—would facilitate harmonized exposure assessment, improve external validity, and enhance the reproducibility of future systematic reviews and meta-analyses. Standardized reporting of probing depth, clinical attachment loss, bleeding on probing, and radiographic bone loss should be encouraged in large prospective cohorts.

Emerging technology-based screening strategies may also contribute to early identification of periodontal inflammation at the community level. For example, recent pilot studies have externally validated artificial intelligence (AI)-based mobile health (mHealth) tools for gingivitis detection among older adults in community settings, demonstrating the feasibility of scalable periodontal screening outside traditional dental clinics [41]. Although such approaches are primarily focused on oral health, they may indirectly support broader preventive strategies by improving early detection and management of periodontal inflammation. However, at present, there is insufficient evidence to support periodontal screening as a cancer prevention strategy.

### Limitations and Interpretation of the Evidence

Several important limitations must be considered when interpreting these findings. First, this meta-analysis is based on only Eight observational studies (primarily cohort designs), which limits statistical power and increases susceptibility to bias. Second, most included studies relied on proxy measures of periodontal disease—such as self-reported periodontitis, tooth loss, or administrative diagnostic codes—rather than standardized clinical periodontal examinations. This variability introduces potential exposure misclassification, which is likely to be predominantly non-differential in prospective cohorts and therefore may attenuate true associations. However, tooth loss in particular represents a non-specific surrogate, as it may result from dental caries, trauma, prosthetic indications, or socioeconomic factors unrelated to periodontal inflammation, thereby reducing specificity and potentially biasing estimates toward the null. Conversely, self-reported periodontal disease may be influenced by health awareness and access to care, which could lead to differential reporting across populations. Such heterogeneity in exposure assessment may partly explain the moderate between-study variability observed.

Third, residual confounding remains a relevant concern, as periodontitis and pancreatic cancer share major risk factors—including smoking, diabetes, obesity, and socioeconomic status—which may not have been fully adjusted for in all cohorts. Fourth, moderate heterogeneity persisted and could not be fully explained by meta-regression analyses, suggesting meaningful methodological variability across studies. Finally, pancreatic cancer is a rare disease; therefore, even the observed relative increase in risk corresponds to a small absolute risk increase. Consequently, the clinical and public health implications of this association should be interpreted cautiously.

Another limitation relates to language restriction, as only English-language publications were included. This may introduce language bias, since relevant studies published in other languages could have been excluded. Although large epidemiological studies on pancreatic cancer are commonly published in English-language journals, the potential impact of language bias should be acknowledged.

Although the present meta-analysis included large population-based cohorts, the number of available studies remains limited, which may affect statistical power and precision. An individual participant data (IPD) meta-analysis would allow for standardized adjustment of confounders, harmonization of periodontal definitions, and more refined subgroup analyses. However, obtaining individual-level data was beyond the scope of the present study. Future collaborative research efforts incorporating IPD approaches may provide more definitive conclusions.

## 5. Conclusions

This meta-analysis suggests a modest statistical association between periodontitis and pancreatic cancer risk; however, the absolute risk increase is small, and the available evidence is insufficient to establish causality. Given the observational nature of the included studies, the limited number of cohorts, and the findings of Trial Sequential Analysis, these results should not be interpreted as supporting clinical or preventive interventions. Further well-designed prospective studies with standardized periodontal assessments and rigorous control of confounding factors are required to clarify the nature of this association.

## Figures and Tables

**Table 1:** Characteristics of the studies included in the meta-analysis.

First Author	Year	Country/Region	Study Design	Sample Size	Cases (Pancreatic Cancer)	Controls	Periodontal Exposure Type	Periodontal Assessment Method	Outcome Definition	Effect Size (95% CI)	Main Confounders Adjusted for	Main Conclusion
Michaud et al. [14]	2007	USA	Prospective Cohort	51,529	216	51,313	Clinical periodontitis	Self-reported history of periodontitis	Pancreatic cancer incidence	HR = 1.64 (95% CI: 1.18–2.28)	Age, smoking (status & pack-years), BMI, diabetes, alcohol intake, physical activity	Periodontitis was significantly associated with an increased risk of pancreatic cancer.
Chang et al. [15]	2016	Taiwan (Asia)	Retrospective Cohort	36,550	341	36,209	Chronic periodontitis	ICD-9 coded periodontal diagnosis	Pancreatic cancer incidence	HR = 1.54 (95% CI: 1.02–2.31)	Age, sex, diabetes, pancreatitis, income, urbanization level	Periodontitis was associated with a significant increase in pancreatic cancer risk.
Yu et al. [16]	2022	UK (Europe)	Prospective Cohort	476,294	4829	471,465	Tooth loss/self-reported poor oral health	Self-reported oral health indicators	Pancreatic cancer incidence	HR = 1.20 (95% CI: 1.12–1.29)	Age, sex, smoking, alcohol, BMI, diabetes, socioeconomic status	Periodontitis increased the risk of pancreatic cancer in adults aged 50–70 years.
Gerlovin et al. [22]	2019	USA	Prospective Cohort	145,000	695	144,305	Self-reported periodontal disease	Questionnaire-based periodontal status	Pancreatic cancer incidence	HR = 1.77 (95% CI: 0.57–5.42)	Age, smoking, BMI, diabetes, alcohol intake, education	A positive association was observed, although it did not reach statistical significance due to low power.
Heikkilä et al. [23]	2018	Finland (Europe)	Nationwide Cohort	68,273	262	68,011	Clinical periodontitis	Nationwide dental treatment registers	Pancreatic cancer incidence	HR = 2.28 (95% CI: 1.31–3.97)	Age, sex, smoking, socioeconomic status, comorbidities	Periodontitis significantly increased pancreatic cancer mortality.
Michaud et al. [18]	2013	USA	Prospective Cohort	73,737	405	73,332	Tooth loss	Questionnaire-based tooth loss history	Pancreatic cancer incidence	RR = 2.14 (95% CI: 1.56–2.92)	Age, smoking, BMI, alcohol intake, diabetes, education	High levels of anti-*P. gingivalis* antibodies were associated with an increased risk of pancreatic cancer.
Fan et al. [17]	2018	China (Asia)	Case–Control Study	1448	724	724	Oral microbiome composition	16S rRNA sequencing	Pancreatic cancer diagnosis	OR = 1.60 (95% CI: 1.25–2.05)	Age, smoking, BMI, alcohol intake, diabetes, education	The presence of *P. gingivalis* significantly increased the risk of pancreatic cancer.
Stolzenberg-Solomon et al. [24]	2003	USA	Prospective Cohort	29,133	373	28,760	Tooth loss	Clinical exams/dental records	Pancreatic cancer incidence	RR = 1.63 (95% CI: 1.24–2.14)	Age, smoking, alcohol intake, BMI	Edentulism was significantly associated with an increased risk of pancreatic cancer.

## Data Availability

The data to support the findings of this study will be available on request from the corresponding author A.I.L.-P.

## Supplementary Materials

The following supporting information can be downloaded at: https://www.mdpi.com/article/10.3390/jcm15083154/s1, Table S1: PRISMA 2020 Checklist; Table S2: GRADE Summary of Findings.
